# Is C-reactive protein associated with influenza A or B in primary care patients with influenza-like illness? A cross-sectional study

**DOI:** 10.1080/02813432.2020.1843942

**Published:** 2020-11-11

**Authors:** Karin Rystedt, Nicolay Jonassen Harbin, Morten Lindbaek, Ruta Radzeviciene, Ronny Gunnarsson, Robert Eggertsen, Christopher C. Butler, Alike W. van der Velden, Theo J. Verheij, Pär-Daniel Sundvall

**Affiliations:** a Region Västra Götaland, Research and Development Primary Health Care, Research and Development Centre Skaraborg, Skövde, Sweden; b Primary Health Care, School of Public Health and Community Medicine, Institute of Medicine, Sahlgrenska Academy, University of Gothenburg, Gothenburg, Sweden; c Centre for Antibiotic Resistance Research (CARe), University of Gothenburg, Gothenburg, Sweden; d Region Västra Götaland, Närhälsan Södra Ryd Health Care Centre, Skövde, Sweden; e Department of General Practice, Antibiotic Center for Primary Care, Institute of Health and Society, University of Oslo, Oslo, Norway; f Mano Seimos gydytojas, Klaipeda, Lithuania; g Region Västra Götaland, Research and Development Primary Health Care, Research and Development Centre Södra Älvsborg, Borås, Sweden; h Region Västra Götaland, Research and Development Primary Health Care, Research and Development Centre Göteborg and Södra Bohuslän, Göteborg, Sweden; i Department of Primary Care Health Services, University of Oxford, Radcliffe Observatory Quarter, Oxford, UK; j Julius Center for Health Sciences and Primary Care, University Medical Center Utrecht, Utrecht, The Netherlands

**Keywords:** C-reactive protein, influenza in humans, point-of-care testing, respiratory tract infections, primary health care

## Abstract

**Objective:**

Identifying influenza A or B as cause of influenza-like illness (ILI) is a challenge due to non-specific symptoms. An accurate, cheap and easy to use biomarker might enhance targeting influenza-specific management in primary care. The aim of this study was to investigate if C-reactive protein (CRP) is associated with influenza A or B, confirmed with PCR testing, in patients presenting with ILI.

**Design:**

Cross-sectional study.

**Setting:**

Primary care in Lithuania, Norway and Sweden.

**Subjects:**

A total of 277 patients at least 1 year of age consulting primary care with ILI during seasonal influenza epidemics.

**Main outcome measures:**

Capillary blood CRP analysed as a point-of-care test and detection of influenza A or B on nasopharyngeal swabs in adults, and nasal and pharyngeal swabs in children using PCR.

**Results:**

The prevalence of positive tests for influenza A among patients was 44% (121/277) and the prevalence of influenza B was 21% (58/277). Patients with influenza A infection could not be identified based on CRP concentration. However, increasing CRP concentration in steps of 10 mg/L was associated with a significantly lower risk for influenza B with an adjusted odds ratio of 0.42 (0.25–0.70; *p*<.001). Signs of more severe symptoms like shortness of breath, sweats or chills and dizziness were associated with higher CRP.

**Conclusions:**

There was no association between CRP and influenza A. Increased concentration of CRP was associated with a lower risk for having influenza B, a finding that lacks clinical usefulness. Hence, CRP testing should be avoided in ILI, unless bacterial pneumonia is suspected.

**Key points**
- Identifying influenza A or B as cause of influenza-like illness (ILI) is a challenge due to non-specific symptoms. There was no association between concentration of CRP and influenza A.
- Increased concentration of CRP was associated with a lower risk for having influenza B, a finding that lacks clinical usefulness.
- A consequence is that CRP testing should be avoided in ILI, unless bacterial pneumonia or similar is suspected.

## Introduction

Influenza-like illness (ILI) refers to patients with acute respiratory tract infection (RTI), having cough and fever [1]. ILI may be caused by influenza A or B, or other viruses. Annual influenza A and B epidemics account for considerable morbidity and mortality [2]. Diagnosing influenza A or B on clinical interpretation alone is difficult due to non-specific symptoms of confirmed influenza infection [3].

PCR testing for influenza is not always a feasible option in primary care. It is either expensive or the sample must be sent to a laboratory for analysis delaying the result. Hence, the availability of cheap PCR testing for influenza as a point of care test (POCT) in primary care is limited. However, CRP is available as a cheap POCT in primary care commonly used to facilitate decisions on whether or not to prescribe antibiotics [4–6].

C-reactive protein (CRP) is an acute-phase protein and the concentration in blood increases in response to inflammation caused by infection, tissue injury or other inflammatory processes [7]. CRP point-of-care testing has been found to reduce antibiotic prescribing in ambulatory care [8]. At the same time a moderately increased concentration of CRP (10–60 mg/L) is a common finding in viral RTIs, mostly with a peak during day 2–4 of the illness [9,10]. Since there are no specific signs or symptoms suggestive for ILI caused by influenza A, B or another virus a clarifying biomarker would be helpful.

There are a few previous studies on CRP, ILI and influenza. A study including 24 emergency department patients with laboratory-confirmed influenza A found that the CRP concentration correlated with severity of symptoms [11]. Other studies have found that among hospitalized influenza patients CRP correlated with hypercytokinemia, acute respiratory distress syndrome, admission to intensive care unit or poor outcome [12–14]. In 79 outpatients with a mix of ILI and other acute RTI, it was found that CRP > 5 mg/L had an odds ratio of 60 (CI 95% 2.7–1400; *p* = .010) for infection with influenza A or B compared to other viruses [15]. Cough, wheezing, absence of leucocytosis and a lower CRP were significant predictors of H1N1 influenza among patients visiting an emergency unit [16]. These previous studies have been small, retrospective, selective for patients with laboratory confirmed influenza, done in hospital settings, or mixed ILI patients with RTI patients without fever.

We, therefore, aimed to investigate whether CRP can predict influenza A or B infection in primary care patients presenting with ILI. We also aimed to examine the association between CRP and various influenza-like symptoms.

## Material and methods

We included patients aged 1 year and older seeking primary care for ILI. The patients were recruited from 30 primary care practices in Lithuania, Norway and Sweden. This cross-sectional study was conducted as a sub study within ALIC^4^E, a multicentre randomised controlled trial (RCT) on the effectiveness of oseltamivir treatment for patients with ILI in primary care [17].

### Inclusion and procedures

Inclusion took place between 27 January 2016 and 4 April 2018 when the prevalence of ILI/influenza in each nations’ epidemiological surveillance passed the threshold for a seasonal epidemic. ILI was defined as a sudden onset of self-reported fever, with at least one respiratory symptom (cough, sore throat or running or congested nose) and one systemic symptom (headache, muscle ache, sweats or chills or tiredness) with a symptom duration of 72 h or less. Patients eligible were those with ILI aged at least one year who could comply with study requirements and who agreed to take an antiviral agent according to randomization. The main exclusion criterion was when the responsible clinician considered urgent hospital admission needed, and the other criteria were described earlier [17]. According to country-specific legislation Lithuania excluded patients who were pregnant, lactating or breastfeeding. All participants were informed of the study, verbally and in writing, and provided written consent before participation. The study was conducted according to the ethical principles of the Declaration of Helsinki.

### Measurements

The two main outcome variables in this sub study were the presence of influenza A or B and the concentration of capillary blood CRP. An oropharyngeal and a nasal swab (COPAN^®^) were taken from those <16 years of age and a nasopharyngeal swab (COPAN^®^) from those ≥16 years of age. The swabs were analysed in the central study lab in Antwerp using a Multiplex RT-PCR for detection of pathogen genes by TaqMan^®^ technology. CRP was taken as capillary blood samples and analysed locally with available point of care equipment for CRP measurement. A case report form was completed upon inclusion covering duration of symptoms, comorbidity, temperature, pulse and the severity of ILI-related symptoms.

The POCT devices used for analysis of CRP concentrations in this study were those used in clinically routine care at the 30 participating primary care practices. Hence, different brands of devices were used. The devices used in Norway and Sweden did not measure values below 5 as the devices used in Lithuania did. CRP concentrations <5 mg/L measured by the devices used in Norway and Sweden were approximated to 3 mg/L.

### Statistical analysis

Four multivariable logistic regression analyses were performed; the first with CRP ≥30 mg/L as dependent variable, the second including only statistically significant variables in the first regression, the third with presence of influenza A as dependent variable and the fourth with the presence of influenza B as dependent variable. In all regression analyses, the independent variables were checked for zero-order correlations using Spearmans rank correlation. A choice was made in case two variables correlated moderately or strongly defined as a statistically significant correlation coefficient above 0.6 or below −0.6. The level of significance was set to 0.05 and IBM SPSS version 25 (IBM SPSS Statistics, Armonk, NY) was used.

#### Factors correlating with CRP ≥ 30 mg/L

Cut-offs between 5.0 and 50 mg/L have been used in previous studies evaluating the use of CRP in ILI [15,16]. We choose a cut-off of 30 mg/L being a compromise between previously used cut-offs. Factors associated with increased CRP concentration as a binary dependent variable with a cut-off at 30 mg/L were tested using multivariable binary logistic regression analysis. All factors statistically significantly associated with a CRP concentration ≥30 mg/L underwent a second and final multivariable binary logistic regression to fine-tune odds ratios.

#### Factors associated with presence of influenza A or B

Factors associated with the presence of influenza A or B were analysed similarly with one model made for influenza A infection and one for influenza B infection. The continuous variable CRP was transformed into a new continuous variable divided by 10 to provide odds ratio for CRP concentration in incremental steps of 10.

## Results

A total of 281 patients were recruited during three consecutive influenza seasons and influenza PCR and CRP were available for 277. Of the 277 patients, 154 (56%) were recruited in Lithuania, 69 (25%) in Sweden and 54 (19%) in Norway. Lithuania participated during the last two influenza seasons while Norway and Sweden participated in all three. The 277 patients consisted of 159 (57%) women and had a mean/median age of 32/30 years (SD 19, interquartile range 18–46), range 1–88. Women (mean age 34 years, SD 19, range 1–88) were slightly older than men (mean age 30 years, SD 19, range 2–76) (*p* = .039). Forty-nine (18%) were children aged <12 years, 215 (78%) were 12–65 years and 13 (4.7%) were >65 years.

### Symptoms, aetiology and CRP concentrations

The three most common symptoms reported as moderate or major severe were fever, feeling generally unwell and having low energy/being tired (Table 1). Overall, patients with a CRP concentration ≥30 mg/L reported more severe individual symptoms. Among the participants 80% (222/277) had CRP concentration <30 mg/L and 20% (55/277) had CRP ≥30 mg/L. The prevalence of influenza A was 44% (121/277 patients) and of influenza B 21% (58/277 patients). Within the influenza A group there were 24 patients with H1N1, constituting 8.7% of all patients. The CRP concentration in ILI patients with different confirmed aetiology had a significant overlap (Table 2).

**Table 1.** Prevalence of moderate or major symptoms in patients with influenza-like illness.

	CRP < 30 mg/L % (*n*)	CRP ≥ 30 mg/L % (*n*)
Fever	84% (186/221)	95% (52/55)
Running or congested nose	58% (128/222)	56% (31/55)
Sore throat	57% (124/218)	64% (34/53)
Headache	73% (156/214)	79% (41/52)
Cough	63% (139/221)	71% (39/55)
Shortness of breath	14% (30/222)	40% (22/55)
Muscle ache and pains	63% (132/209)	79% (42/53)
Sweats or chills	62% (137/222)	87% (48/55)
Diarrhoea	2.7% (6/221)	3.6% (2/55)
Nausea and/or vomiting	5.4% (12/222)	18% (10/55)
Abdominal pain	5.5% (12/219)	13% (7/53)
Low energy/tired	79% (176/222)	94% (50/53)
Not sleeping well	50% (111/221)	65% (35/54)
Dizziness	20% (42/208)	48% (25/52)
Feeling generally unwell	85% (183/216)	96% (49/51)
Overall symptom duration^a^		
≤24 h	32% (71/222)	18% (10/55)
>24–48 h	35% (77/222)	42% (23/55)
>48–72 h	33% (74/222)	40% (22/55)

^a^Duration from first symptom until inclusion in study.

**Table 2.** C-reactive protein (CRP) in patients with influenza-like illness (ILI).

	Patients	CRP (mg/L) median	Interquartile range	Min–max
Influenza A	44% (121/277)	13	6.0–27	1–173
H1N1	8.7% (24/277)	24	9.5–43	1–173
Influenza B	21% (58/277)	5.0	3.0–11	0–41
Not influenza A or B	35% (98/277)	16	3.0–34	0–210
ILI regardless of aetiology	100% (277/277)	10	3.0–25	0–210

### Influenza A or B and their association with CRP

None of the independent variables correlated significantly with each other when tested with Spearman rank correlation. Hence, all independent variables could be used for multivariable regression modelling. Patients reporting moderate or major severity of symptoms such as shortness of breath, sweats or chills and dizziness were more likely to have a CRP concentration ≥ 30 mg/L (Table 3). CRP ≥ 30 mg/L was associated with a lower risk for influenza B with an adjusted odds ratio of 0.12 (0.030–0.47; *p* = .0025) (Table 3). Increased CRP concentrations were also associated with illness duration 48–72 h (Table 3).

**Table 3.** Factors associated with C-reactive protein (CRP) ≥ 30 mg/L in patients with influenza-like illness.

	Adjusted odds ratio all variables^a^ (95% CI; *p* value)	Adjusted odds ratio authors preference^b^ (95% CI; *p* value)
Not influenza (reference)	1.0	1.0
Influenza A	0.56 (0.24–1.4; *p*=.20)	0.63 (0.30–1.3; *p*=.21)
Influenza B	0.059 (0.0064–0.54; *p*=**.012**)	0.12 (0.030–0.47; *p*=**.0025**)
Increasing age in years	1.0 (0.97–1.0; *p* = 1.0)	
Female gender	0.78 (0.33–1.8; *p*=.57)	
Duratio*n* < 24 h (reference)	1.0	1.0
Duration 24–48 h	3.1 (1.0–9.1; *p*=**.042**)	2.2 (0.89–5.5; *p*=.087)
Duration 48–72 h	2.8 (0.91–8.3; *p*=.072)	2.9 (1.1–7.2; *p*=**.027**)
Increasing pulse of one beat	0.99 (0.96–1.0; *p*=.58)	
Increasing body temperature of one degree Celsius	1.6 (0.94–2.7; *p*=.085)	
Your health today^c^	0.98 (0.96–1.0; *p*=.11)	
Perceived fever^d^	2.3 (0.42–13; *p*=.34)	
Running or congested nose^d^	0.69 (0.28–1.7; *p*=.44)	
Sore throat^d^	1.2 (0.48–3.0; *p*=.69)	
Headache^d^	0.63 (0.22–1.8; *p*=.39)	
Cough^d^	0.62 (0.24–1.5; *p*=.30)	
Shortness of breath^d^	4.0 (1.4–12; *p*=**.012**)	3.1 (1.4–6.7; *p* = **0.0037**)
Muscle ache and pains^d^	1.5 (0.52–4.5; *p*=.44)	
Sweats or chills^d^	3.3 (1.0–11; ***p*=.044**)	4.2 (1.6–11; *p* = **0.0036**)
Low energy/tired^d^	1.8 (0.36–8.6; *p*=.48)	
Not sleeping well^d^	0.77 (0.31–1.9; *p*=.57)	
Dizziness^d^	3.1 (1.1–8.5; *p*=**.027**)	3.0 (1.5–6.3; *p* = **0.0029**)
Feeling generally unwell^d^	1.2 (0.19–7.4; *p*=.86)	

^a^224 included in analysis. Nagelkerke R square 0.35. Area under the curve (95% CI; *p* value): .84 (0.78–0.91; <.001).

^b^260 included in analysis. Nagelkerke R square 0.30. Area under the curve (95% CI; *p* value): .82 (0.76–0.88; <.001).

^c^EQ-VAS EuroQol Group: 0 = worst possible health, 100 = best possible health. Odds ratio given for an increase of one step.

^d^Moderate or major symptoms.

Statistically significant findings at 5% level are bold.

The multivariable model predicting influenza B was better than the corresponding model trying to predict influenza A with a Nagelkerke R square 0.39 and area under the ROC curve of 0.86 (95% CI 0.81–0.92; *p*<.001) (Table 4). The model predicting influenza B infection also showed a correlation between influenza B infection and an incremental increase of CRP in steps of 10 mg/L with an odds ratio of 0.42 (Table 4).

**Table 4.** C-reactive protein (CRP) and other factors to differentiate between influenza A and B vs other causes of influenza-like illness (ILI).

	influenza A^a^			influenza B^b^		
	Adjusted OR^c^	95% CI	*p* Value	Adjusted OR^d^	95% CI	*p* Value
CRP (mg/L)^e^	1.0	0.91–1.2	.61	0.42	0.25–0.70	**<.001**
Increasing age in years	1.0	0.98–1.0	.84	0.99	0.97–1.0	.52
Female gender	1.4	0.78–2.6	.25	0.53	0.21–1.3	.18
Duration ref <24 h	1.0			1.0		
Duration 24–48 h	0.85	0.41–1.7	.65	0.43	0.13–1.4	.16
Duration 48–72 h	0.71	0.34–1.5	.36	2.2	0.83–6.1	.11
Increasing body temperature of one degree Celsius	1.1	0.76–1.6	.60	1.3	0.72–2.3	.40
Increasing pulse of one beat	1.0	0.98–1.0	.56	0.97	0.94–1.0	.22
Your health today^f^	0.99	0.97–1.0	.091	1.0	0.98–1.0	.61
Perceived fever^g^	2.1	0.82–5.7	.12	1.6	0.42–6.2	.48
Running/congested nose^g^	1.2	0.63–2.2	.63	0.69	0.29–1.6	.40
Sore throat^g^	0.53	0.29–0.97	**.038**	0.79	0.33–1.9	.59
Headache^g^	0.85	0.41–1.7	.65	2.8	0.87–9.3	.085
Cough^g^	1.4	0.75–2.7	.28	3.4	1.2–9.1	**.017**
Shortness of breath^g^	1.3	0.52–3.0	.61	0.16	0.028–0.89	**.036**
Muscle ache and pains^g^	1.6	0.82–3.2	.17	0.94	0.37–2.4	.89
Sweats or chills^g^	1.3	0.61–2.6	.52	0.75	0.25–2.3	.62
Low energy/tired^g^	0.48	0.18–1.3	.14	1.5	0.34–6.7	.59
Not sleeping well^g^	1.2	0.63–2.3	.56	0.57	0.22–1.5	.25
Dizziness^g^	0.54	0.25–1.2	.12	4.3	1.3–14	**.014**
Feeling generally unwell^g^	1.8	0.62–5.0	.28	0.81	0.19–3.4	.77

^a^Influenza A versus all other etiologies (influenza B and not influenza).

^b^Influenza B versus all other etiologies (influenza A and not influenza).

^c^224 included in analysis (104 with influenza A and 120 with other etiology). Nagelkerke R square 0.14. Area under the curve (95% CI; *p* value): 0.68 (0.61–0.75; <.001).

^d^224 patients included in analysis (43 with influenza B and 181 with other etiology). Nagelkerke R square 0.39. Area under the curve (95% CI; *p* value): 0.86 (0.81–0.92; <.001).

^e^Incremental steps of ten.

^f^EQ-VAS EuroQol Group: 0 = worst possible health, 100 = best possible health. Odds ratio given for an increase of one step.

^g^Moderate or major symptoms.

Statistically significant findings at 5% level are bold.

## Discussion

We found that CRP ≥ 30 mg/L was associated with a lower risk of influenza B infection. We did not find an association between CRP and influenza A. This study is to our knowledge the first to investigate the potential usefulness of CRP testing to predict presence of influenza virus A or B, or no influenza virus, in ILI patients presenting in primary care.

### Strengths and limitations

The strength of this study is the pragmatic design reflecting typical clinical practice and the variety of patients with respect to age, ILI severity and comorbidities having sought primary care with broad-spectrum symptoms of ILI. They were prospectively recruited based on their symptoms and not retrospectively because of a previous laboratory finding of influenza A or B. The results can therefore be meaningful and applicable in other primary care settings.

One limitation was that we lacked multiple CRP tests showing the course over time as we know that the concentration of CRP can change rapidly [18]. Another limitation is that we did not have data on other aetiological agents than influenza A and B. We cannot rule out that a clinically missed diagnosis of bacterial pneumonia could explain single high CRP concentrations.

### CRP, symptoms and influenza A or B

The finding that CRP ≥ 30 mg/L was associated with a lower risk for having influenza B has not been reported earlier, but is consistent with the understanding of differences between influenza B and A [19]. The low median CRP concentration we found in patients with influenza B is in accordance with findings in a recent study of influenza B-associated myositis in children. In 47 influenza B positive children the median CRP was 3.4 mg/L (range 0.3–19 mg/L) [20].

We did not find an association between the concentration of CRP and influenza A. This finding is discordant to earlier findings by Cinemre et al. who found that CRP ≥ 5 mg/L was a positive predictor for influenza A and/or B infection [15]. One reason for this discrepancy can be the difference in cut-off level, Cinemre et al. used CRP ≥ 5 mg/L as cut-off and we used CRP ≥ 30 mg/L. Another reason might be the difference in the selection of patients. In our study, we focused on ILI patients during seasonal influenza epidemics, and self-reported fever was an inclusion criterion. Cinemre et al. had wider inclusion criteria and most patients were having acute respiratory infection, which might have been milder disease without fever. As expected, the median CRP was lower in our study compared to a study of hospitalized patients with influenza [21].

Influenza A was associated with a lower risk of sore throat (Table 4). This is probably explained by other aetiological agents than influenza A being more prone to cause sore throat as part of the RTI.

## Conclusion

The main finding was that CRP ≥ 30 mg/L was associated with a lower risk for having influenza B. However, there is no obvious clinical usefulness of this knowledge. There was no association between CRP concentration and influenza A. Hence, we don’t recommend CRP to be used for diagnosing influenza A or B. It should be noted that this study did not evaluate the use of CRP testing in diagnosing pneumonia or other serious illness as a differential diagnosis, or complication to influenza infection.
